# Association between serum γ-Glutamyltransferase and the risk of cervical cancer: Evidence from the national health and nutrition examination survey

**DOI:** 10.1371/journal.pone.0339001

**Published:** 2026-01-02

**Authors:** Lu Qiu, Yuanfu Xie, Yimin Li, Jianli Huang, Youjia Wang, Yanhong Zhuo

**Affiliations:** Department of Radiation Oncology, Zhangzhou Affiliated Hospital of Fujian Medical University, Zhangzhou, China; Universidad Autonoma de Yucatan, MEXICO

## Abstract

**Background:**

The principal cause of cervical cancer is sustained high-risk human papillomavirus (HPV) infection. However, some cases remain unrelated to HPV infections. Current HPV screening methods have difficulties identifying HPV-negative patients and implementing them in low-resource settings. Therefore, it is important to explore easily accessible and low-cost biomarkers to optimize the current cervical cancer screening strategies.

**Methods:**

This cross-sectional study included 6998 women from the US National Health and Nutrition Examination Survey (NHANES), among whom 147 had self-reported cervical cancer. Serum γ – Glutamyltransferase (GGT) was employed as a continuous variable (naturally logarithmically transformed) and a categorical variable (≥50 U/L versus <50 U/L), and multivariate logistic regression models were utilized to progressively adjust for demographic features, sexual history, and clinical behaviors. The restricted cubic spline approach was adopted to further explore the dose-response relationship between GGT levels and cervical cancer to depict the potential nonlinear trends between them in a more elaborate manner. We also conducted subgroup and sensitivity analyses to ensure reliability of our results. This included multiple imputation of missing data and adjustment for crucial covariates, such as body mass index (BMI) and tobacco exposure, to comprehensively evaluate the impact of different factors on the outcomes. Ultimately, through mediation analysis, we probed the mediating function of tobacco exposure in the association between GGT levels and cervical cancer, aiming to obtain a more profound understanding of the underlying mechanisms of this health-related relationship.

**Results:**

Serum GGT levels are positively correlated with cervical cancer risk. For each log unit increase in GGT levels, there was a 31% increased risk of cervical cancer (OR = 1.31, 95%CI: 1.01–1.70, P = 0.041) in the model adjusted for multiple risk factors. When the GGT level reached or exceeded 50 U/L, the risk increased by 76% (OR = 1.76, 95%CI: 1.04–2.98, P = 0.034). This tendency was consistent across several crucial subgroups, particularly among HPV-negative women (OR = 1.36, 95%CI: 1.01–1.84), suggesting that GGT may have some significance in different populations. The results of the sensitivity analyses demonstrated that the main findings remained robust, even when multiple imputations were employed to handle missing data. Nevertheless, the significant association between GGT and cervical cancer weakened when tobacco exposure was further adjusted, indicating that tobacco use might be involved. Mediation analysis further showed that the overall impact of GGT on cervical cancer was statistically significant (β = 0.0014; 95%CI: 0.0001–0.0020; P = 0.046), and approximately 18.15% of the impact was accounted for by tobacco exposure (ACME: β = 0.0003, 95%CI: 0.0001–0.0004, P < 0.001), suggesting a partial mediating role.

**Conclusion:**

Elevated serum GGT levels are associated with a heightened risk of cervical cancer even in HPV-negative patients. Although this association is partly mediated by tobacco exposure, GGT may still play a role in cervical cancer via nontobacco routes. The potential application of GGT as an auxiliary biomarker requires further validation in prospective studies.

## Introduction

Cervical cancer has an annual incidence of 604 000 cases and 342 000 deaths, approximately 85% of which occur in low- and middle-income countries [1]. The primary cause of cervical cancer is a continuous infection with high-risk human papillomavirus (HPV) [2]. The tumor suppressor proteins E6 and E7 produced by HPV can degrade tumor suppressor proteins p53 and Rb, respectively, which are key causes of cervical cancer [3,4]. However, there are still some cases of HPV-negative cervical cancer (e.g., gastric adenocarcinoma and endometrioid adenocarcinoma), suggesting the existence of non-HPV-related oncogenic pathways in the development of cervical cancer [5,6]. If we had relied only on HPV screening, these individuals may have been missed. In addition, large-scale population-based HPV testing will face barriers in low-resource settings [7,8]. In this context, the search for an easily accessible and low-cost biomarker may help optimize current cervical cancer screening protocols, especially in resource-limited settings and HPV-negative populations.

Serum γ-glutamyltransferase (GGT), an enzyme predominantly located on the cell membranes, is critical for regulating extracellular glutathione metabolism [9]. This catalytic process can produce reactive oxygen species (ROS) under specific conditions, making GGT not only a key mediator of oxidative stress but also an important indicator of the oxidative load of the body [10]. Given that oxidative stress and chronic inflammation are common pathological bases of various chronic diseases, including cancer, diabetes, and cardiovascular diseases, this dual feature of GGT makes it a potential biomarker for assessing the risk of such diseases [11]. Oxidative stress and chronic inflammation are widely confirmed as important factors that drive tumorigenesis and development [12]. As a key link in the process of oxidative stress, we postulated that GGT may drive cervical carcinogenesis through oxidative stress modulation, and its mechanism may be synergistic with or independent of HPV infection. Specifically, GGT-derived ROS have been shown to activate pro-cancer signaling pathways such as NF-κB [13]. The NF-κB pathway is often dysfunctional in cervical cancer and can promote tumor cell survival, proliferation, and metastasis [14–16]. Based on these findings, we postulate that elevated serum GGT levels may increase the susceptibility of women to cervical malignancy, regardless of HPV serostatus, by activating the redox homeostasis pathway.

In recent years, GGT has drawn a growing interest in the field of cancer research. GGT has been widely regarded as an independent predictor of cancer risk in studies of head and neck tumors as well as tumors of the digestive, genitourinary, and respiratory systems [17–20]. Regarding common malignant tumors in women, Seol et al. found a significant association between increased serum GGT levels and the risk of breast cancer [21]. A longitudinal study by Han et al. also showed that increased GGT activity was closely related to the occurrence of endometrial cancer [22]. Several subsequent studies have provided supportive evidence for a significant positive correlation between circulating GGT levels and the risk of invasive cervical cancer. No statistically significant relationship was detected between GGT levels and cervical intraepithelial neoplasia grade III (CIN III) [23]. Stephan et al. also explored the potential value of GGT as a biomarker reflecting the balance of apoptosis in predicting tumor staging and prognosis in cervical cancer. Studies have found that elevated GGT levels often occur in the later stages of cervical cancer, suggesting that they may be related to disease progression; however, there is still a lack of clear prognostic cut-off values [24]. Collectively, these studies indicate that GGT may be a potential reference indicator for various malignant tumors. Currently, research regarding the relationship between GGT levels and cervical cancer remains in its initial phase, and relevant evidence is inadequate. There is still ample scope for us to conduct in-depth exploration to comprehensively comprehend the scientific value and health implications underlying this association.

To address the unresolved questions in the current study, we comprehensively investigated the potential relationship between persistently elevated GGT levels and the risk of cervical cancer using data from the US National Health and Nutrition Examination Survey (NHANES). We postulated that elevated GGT levels might be linked to a heightened risk of cervical cancer. It is anticipated that this investigation can offer new leads and substantial support for the early detection and individualized prevention strategies of cervical cancer, and also bring promise to the health safeguarding of more women.

## Materials and methods

### Data sources and design

We examined the NHANES data from 2003 to 2016. NHANES is a nationally representative survey conducted by the Centers for Disease Control and Prevention (CDC) with the aim of comprehending the health and nutritional status of the non-hospitalized population in the United States [25]. Simultaneously, we meticulously referred to the Guidelines for Strengthening the Reporting of Observational Studies in Epidemiology (STROBE) to conduct research reports and strive to present the research findings in a clear and transparent manner [26], thereby contributing a reliable and responsible effort to scientific communication.

### Ethics declarations

The National Health and Nutrition Examination Survey (NHANES) got the approval by the NCHS Ethics Review Board (Protocols #98–12, #2005–06, and #2011–17). All the participants provided written informed consent.

This secondary analysis of anonymized and publicly accessible data was reviewed and approved by the Ethics Committee of the Zhangzhou Affiliated Hospital of Fujian Medical University. The committee consented to exempt this study from further review as it utilized de-identified secondary data.

### Study population

This observational study was a cross-sectional analysis based on the NHANES dataset [27], and the research samples were collected from the NHANES survey data from 2003 to 2016. As per the NHANES protocol, the cancer questionnaire targeted adults aged ≥20 years [28] and high-risk HPV testing was restricted to women aged 18–59 years [29]. To create a uniform cohort in which all participants had complete data on both the cancer questionnaire and high-risk HPV status, the analysis was limited to women aged 20–59 years old. This age range overlaps with the range applicable to these measures across the NHANES data cycles.

### Participant selection

**Fig 1:**
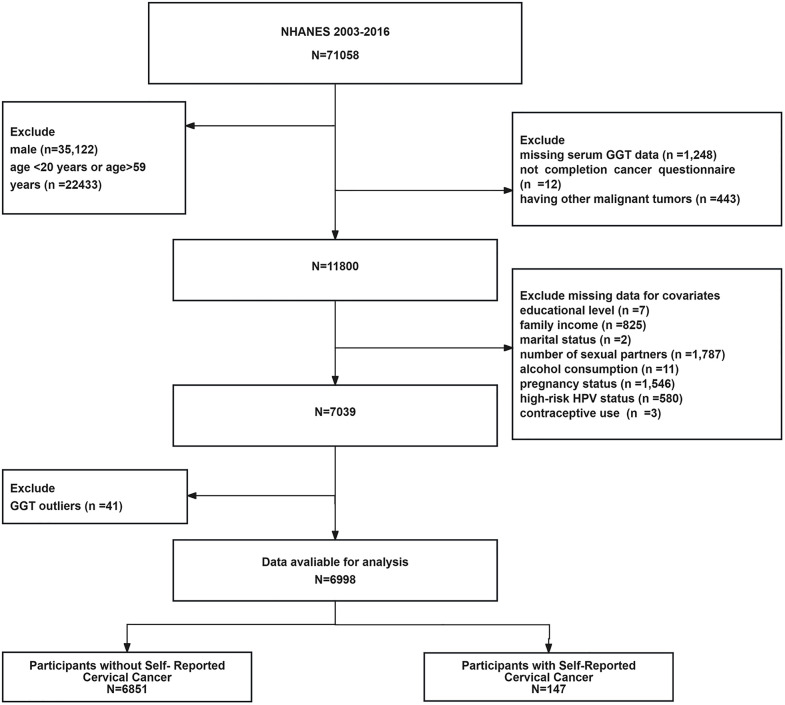
Flow diagram of participants for the National Health and Nutrition Examination Survey 2003-2016. **Note:** The age range of 20–59 years was selected because it represents an overlapping cohort for which both cancer history (available for adults ≥20 years) and high-risk HPV data (available for women 18–59 years) were available in the NHANES.

From 2003 to 2016, 71,058 participants completed the NHANES survey. Participants who met the following criteria were included: women aged 20–59 years who completed the cancer questionnaire and had available serum GGT data. The exclusion criteria were as follows: male (n = 35,122), not within the age range of 20–59 years (n = 22,433), missing serum GGT test data (n = 1,248), no completion of the cancer questionnaire (n = 12), having a history of other malignant tumors (n = 443), educational level (n = 7), household income (n = 825), marital status (n = 2), number of sexual partners (n = 1,787), alcohol consumption (n = 11), pregnancy status (n = 1,546), high-risk HPV infection status (n = 580), and contraceptive use (n = 3). GGT outliers were defined a priori as values below the 0.5th percentile or higher than the 99.5th percentile of the distribution [30,31]. The participants who met this criterion (n = 41) were eliminated to ensure the soundness of the results. The initial sample comprised 71,058 participants. Inclusion and exclusion criteria were applied systematically, and cases with missing covariate values were excluded because of incomplete data. Patients with abnormal GGT levels were excluded from this study. The final sample size obtained in this process was 6,998 individuals, 147 of whom had self-reported cervical cancer. The participant inclusion and exclusion criteria are illustrated in the flowchart (see **Fig 1**).

GGT outliers referred to participants whose γ-glutamyltransferase (GGT) test results were below the 0.5th percentile or above the 99.5th percentile (n = 41).

### Measurement of GGT

From the detailed records in the NHANES laboratory methods [32], we obtained the complete methodological details of the sample collection process, storage conditions, and analysis procedures. GGT levels of GGT was measured directly from the laboratory samples. To guarantee the precision of the measurement results, laboratory researchers strictly controlled the cold chain conditions at all stages from sample collection and transportation to the final analysis. In the principle of detection, they took advantage of the specific reaction of GGT, which catalyses the transfer of γ-glutamyl groups to glycine and generates p-nitroaniline. The dynamic change in absorbance of this product was continuously monitored at 410 nm using an automated spectrophotometer, and the rate of absorbance change was directly converted to GGT activity values by comparison with a pre-calibrated standard.

### The assessment of cervical cancer

Cervical cancer cases were identified via a positive response to the NHANES inquiry: “Has a doctor or any other healthcare professional ever told you that you had cervical cancer?” We did not have access to histopathology reports, cancer registry linkage, or date of diagnosis; therefore, all cases were self-reported prevalent cases rather than incident histologically confirmed cervical cancers [33,34]. Data were collected during household interviews in accordance with the NHANES procedures [35].

### Evaluation of Covariates

When evaluating covariates, we aimed to gain a clearer understanding of the independent relationship between serum GGT level and cervical cancer. To this end, we systematically included and adjusted for potential confounders that could have influenced the results, based on clinical and epidemiological evidence. We looked at a range of structural and social factors that affect health, Included were race/ethnicity (categorized as non-Hispanic white, non-Hispanic African American, Mexican American, and other races [36]), education level (up to 9th grade or lower, 10th to 12th grade or higher [37]), and marital status (married or living with a partner vs. Living alone [38]) and socioeconomic status (according to poverty income ratio PIR: low income ≤1.3, middle income 1.3–3.5, high income >3.5 [39]). To varying degrees, these factors may affect people’s access to health services and cancer risk [40], we uniformly adjusted for these factors in our analyses to reflect the true associations more fairly and comprehensively. Given the important influence of sexual and reproductive factors on HPV-related lesions, we also included information on age at first sexual intercourse, number of sexual partners, age at menarche, number of pregnancies, and specifically prioritized adjustment for high-risk HPV infection status (determined by oncogenic genotype testing [41]) as a core background factor for cervical cancer development. In addition, several lifestyle factors may also influence GGT levels [42,43]; therefore, we also considered contraceptive use (based on self-reported history of use [44]) and drinking habits (defined as drinking more than 12 drinks per year [45]) to capture potential pathways of effect more meticulously. Finally, in sensitivity analyses, we further explored body mass index BMI (classified as normal (BMI ≤ 25 kg/m^2^), overweight (25 < BMI < 30 kg/m^2^), and obesity (BMI ≥ 30 kg/m^2^ [46]) and tobacco exposure (defined as serum cotinine level ≥0.05 ng/mL [47]). These indicators may interfere with research results through mechanisms such as chronic inflammation and oxidative stress [48,49]. We hope that, through this comprehensive consideration, the research results will be more robust, credible, and relevant to the complexity of the real world.

### Statistical analysis methods

Data are presented as mean ± standard deviation (SD) for continuously distributed variables that conform to a normal distribution, median and interquartile range (IQR) for skewed variables, and frequency (percentage) for categorical data. For group comparisons, the independent samples t-test or Mann-Whitney U test was used for continuous variables, depending on whether the distribution was normal, and the chi-square test was used for categorical variables. The exact p-values are presented in the results section.

Because of its skewed distribution, GGT values were log-transformed for the multivariate analyses. We initially established a relationship between GGT levels and cervical cancer by using a set of logistic regression models. Starting with an unadjusted baseline (Model 1), we sequentially incorporated demographic factors (age, race/ethnicity, education, family income, and marital status; Model 2), and sexual and reproductive history (number of pregnancies, age at menarche, number of sexual partners, and age at first intercourse) were factors (high-risk HPV, contraceptive use, and alcohol consumption; Model 4). Subsequently, we characterized the dose – response pattern using restricted cubic spline (RCS) regression. A spline was configured by specifying four knots at the 5th, 35th, 65th, and 95th percentiles, respectively. The model was fitted using covariate adjustments to produce a smooth curve.

We performed exploratory subgroup analyses to formulate hypotheses for future research on the consistency of the associations across crucial demographic and clinical strata. Given the exploratory nature of these subgroup analyses, p-values were not adjusted for multiple comparisons to prevent an increase in type II errors. Thus, the results of the subgroup analyses should be regarded as hypothesis generating. To conduct a formal test for effect modification, we incorporated a multiplicative interaction term between the log-transformed GGT levels and each subgroup variable into the fully adjusted model. The statistical significance of the interaction was evaluated using the likelihood ratio test.

In the primary analysis, missing data were handled using listwise deletion, whereby participants with incomplete records on key study variables were excluded from the analytic sample. In the first sensitivity analysis, we used multiple imputations to assess the possible effects of missing data on covariates. The analysis included 11,800 participants aged 20–59 years who had complete serum GGT data and completed the cancer questionnaire. We first performed five multiple imputations for missing covariates to make the best use of the available information, then removed outliers for GGT (n = 67), and finally included 11,733 participants in the analysis (see **S1 Fig**). The association between serum GGT level and cervical cancer was reassessed using multivariate regression models to test the stability of the results. In the second sensitivity analysis, we explored whether BMI and tobacco exposure might have influenced our conclusions. The analysis was based on a complete case dataset. After excluding participants with missing covariate information (n = 7039) in the main analysis, we further excluded those with missing BMI data (n = 26), missing serum cotinine test results (n = 1), and abnormal GGT values (n = 41). A total of 6971 participants were enrolled (see **S1 Fig**). We constructed two new models from the fully adjusted model used in the primary analysis (Model 4): Model 9 with categorical variables for BMI and Model 10 with measures related to tobacco exposure. Subsequently, the association between GGT levels and cervical cancer was assessed by multivariate regression analysis using a method consistent with the main analysis. Specific definitions of these two covariates are detailed in the covariate assessment section.

To establish whether tobacco exposure represented a confounding factor or a mediating effect, we performed a mediation analysis. In the designated mediation model, log-transformed GGT levels functioned as the independent variable, cervical cancer status as the outcome variable, and serum cotinine level (a continuous indicator of tobacco exposure) as the mediator variable. All confounders incorporated in the primary fully adjusted model (Model 4) were adjusted for as covariates in the mediator models. The significance of the average causal mediation effect (ACME, also referred to as the indirect effect), average direct effect (ADE), and total effect was evaluated via nonparametric bootstrapping with 1000 simulations to obtain reliable 95% confidence intervals. The mediated proportion was calculated as the ratio of ACME to the Total Effect.

All statistical analyses were performed using the R software (version 4.2.2; R Foundation) and the Free Statistics platform (version 2.1). A two-tailed p-value of less than 0.05 was regarded as statistically significant.

## Results

### Characteristics of the study population

The baseline Characteristics of the 6,998 participants (147 with cervical cancer and 6,851 without) are presented in **Table 1**. Participants with cervical cancer had significantly higher median serum GGT levels (17.0 U/L [IQR 12.0–28.0]) than those without (16.0 U/L [IQR 12.0–24.0]; P = 0.030). Although cases were on average 1.5 years older, the difference was not statistically significant (41.9 ± 10.4 vs. 40.4 ± 10.6 years; P = 0.075). Significant differences were observed in several demographic and behavioral factors. A greater proportion of patients were non-Hispanic white (72.8% vs. 41.2%; P < 0.001), had 9th–12th grade education (48.3% vs. 36.8%; P = 0.017), and had a low family income (51.0% vs. 34.6%; P < 0.001). Regarding reproductive health, patients reported an earlier median age at first sexual intercourse (16 vs. 17 years; P < 0.001) and had a higher prevalence of high-risk HPV infection (29.9% vs. 22.3%; P = 0.028) and contraceptive use (89.1% vs. 77.9%; P = 0.001).

**Table 1:** Baseline characteristics of the study participants in the NHANES 2003-2016 cycles.

Variables	All participants (n = 6998)	Non-cases (n = 6851)	Cases (n = 147)	P value
**Age, years, Mean ± SD**	40.4 ± 10.6	40.3 ± 10.6	41.9 ± 10.4	0.075
**Race and ethnicity, n (%)**				< 0.001
Non-Hispanic White	2931 (41.9)	2824 (41.2)	107 (72.8)	
Non-Hispanic Black	1575 (22.5)	1562 (22.8)	13 (8.8)	
Mexican-American	1290 (18.4)	1280 (18.7)	10 (6.8)	
Other race^a^	1202 (17.2)	1185 (17.3)	17 (11.6)	
**Education level, n (%)**				0.017
<9th grade	451 (6.4)	444 (6.5)	7 (4.8)	
9th-12th grade	2595 (37.1)	2524 (36.8)	71 (48.3)	
>12th grade^b^	3952 (56.5)	3883 (56.7)	69 (46.9)	
**Family income** ^**c**^ **, n (%)**				< 0.001
Low income	2443 (34.9)	2368 (34.6)	75 (51.0)	
Medium income	2439 (34.9)	2395 (35.0)	44 (29.9)	
High income	2116 (30.2)	2088 (30.5)	28 (19.0)	
**Marital status, n (%)**				0.003
Married or living with a partner	4571 (65.3)	4492 (65.6)	79 (53.7)	
Living alone^d^	2427 (34.7)	2359 (34.4)	68 (46.3)	
**High-risk HPV status** ^**e**^ **, n (%)**				0.028
Negative	5426 (77.5)	5323 (77.7)	103 (70.1)	
Positive	1572 (22.5)	1528 (22.3)	44 (29.9)	
**Contraceptive use** ^**f**^ **, n (%)**				0.001
No	1532 (21.9)	1516 (22.1)	16 (10.9)	
Yes	5466 (78.1)	5335 (77.9)	131 (89.1)	
**Alcohol consumption** ^**g**^ **, n (%)**				0.078
No	2381 (34.0)	2341 (34.2)	40 (27.2)	
Yes	4617 (66.0)	4510 (65.8)	107 (72.8)	
**Number of sexual partners,** **Median (IQR)**	1.0 (0.0, 5.0)	1.0 (0.0, 5.0)	2.0 (0.0, 9.0)	0.355
**Age at first sexual intercourse,** **Median (IQR)**	17.0 (15.0, 19.0)	17.0 (15.0, 19.0)	16.0 (15.0, 17.0)	< 0.001
**Number of pregnancies,** **Median (IQR)**	3.0 (2.0, 4.0)	3.0 (2.0, 4.0)	3.0 (2.0, 4.0)	0.009
**Age at first menstruation,** **Median (IQR)**	13.0 (12.0, 14.0)	13.0 (12.0, 14.0)	12.0 (11.0, 13.0)	0.061
**GGT, U/L, Median (IQR)**	16.0 (12.0, 24.0)	16.0 (12.0, 24.0)	17.0 (12.0, 28.0)	0.030

**Note:** Data are presented as mean ± standard deviation, median (IQR), or number (percentage). P-values for continuous variables were derived from the independent samples t-test (normally distributed) or the Mann-Whitney U test (skewed distribution); p-values for categorical variables were derived from the chi-square test.

Abbreviations: GGT, γ-glutamyltransferase; HPV, human papillomavirus; NHANES, National Health and Nutrition Examination Survey; PIR, poverty income ratio.

**Footnotes:**

^a.^ The “Other” race/ethnicity category includes Hispanic and other non-Hispanic races (including non-Hispanic multiracial).

^b.^ The 12th grade education category included college and college graduates or above.

^c.^ Family income was classified into three levels according to PIR: low (PIR ≤ 1.3), medium (1.3 < PIR ≤ 3.5), and high (PIR > 3.5).

^d.^ The “living alone” marital status category includes widowed, divorced, separated, and never-married individuals.

^e.^ High-risk HPV infection was defined as detection of any carcinogenic genotype (16, 18, 31, 33, 35, 39, 45, 51, 52, 56, 58, 59, 66, or 68).

^f.^ Contraceptive use was ascertained by a positive response to the question, ‘Have you ever used birth control pills?’

^g.^ Alcohol consumption was determined as a positive response to the question,‘In any single year, have you consumed at least 12 drinks of any kind of alcoholic beverage?’

### Multivariable regression analyses

We evaluated the relationship between natural log-transformed GGT levels and cervical cancer by using progressively adjusted logistic regression models (see **Table 2**). Even in the unadjusted model (Model 1), we detected a positive association, where each unit increase in ln(GGT) was associated with 43% higher odds of cervical cancer (OR (odds ratio) = 1.43, 95% CI (confidence interval): 1.12–1.83; P = 0.005). The models were adjusted for an expanding set of covariates, starting from socio-demographics (Model 2: OR = 1.35, 95% CI: 1.05–1.75; P = 0.021) and then to sexual/reproductive history (Model 3: OR = 1.31, 95% CI:1.01–1.70; P = 0.038). The association showed a slight reduction in magnitude yet retained statistical significance. The positive association between GGT levels and cervical cancer remained significant in the final model (Model 4: OR = 1.31, 95% CI: 1.01–1.70; P = 0.041). Additionally, based on the clinical threshold of 50 U/L GGT [50], the model was gradually adjusted once more, and the results of the multivariate regression analysis still verified the robustness of the association between them. Participants with GGT levels ≥50 U/L had a significantly increased cervical cancer risk compared to those below this threshold across all multivariate models: Model 2 (OR = 1.84, 95% CI: 1.09–3.09, P = 0.022), Model 3 (OR = 1.75, 95% CI: 1.03–2.95, P = 0.037), and Model 4 (OR = 1.76, 95% CI: 1.04–2.98, P = 0.034).

**Table 2:** Multivariable logistic regression analyses of the association between serum γ-glutamyltransferase and cervical cancer.

Variable	Event, n/N (%)	Model 1 OR (95% CI)	P value	Model 2 OR (95% CI)	P value	Model 3 OR (95% CI)	P value	Model 4 OR (95% CI)	P value
**GGT(log)**	147/6998 (2.1)	1.43 (1.12–1.83)	0.005	1.35 (1.05–1.75)	0.021	1.31 (1.01–1.70)	0.038	1.31 (1.01–1.70)	0.041
**GGT groups**									
GGT < 50U/L	129/6560 (2.0)	1(Reference)	—	1(Reference)	—	1(Reference)	—	1(Reference)	—
GGT ≥ 50U/L	18/438 (4.1)	2.14 (1.29–3.53)	0.003	1.84 (1.09–3.09)	0.022	1.75 (1.03–2.95)	0.037	1.76 (1.04–2.98)	0.034

**Note:** Data are presented as OR with 95% CI intervals GGT levels were analyzed both as a continuous variable (for each 1 – unit increase in the natural log-transformed value) and as a categorical variable.

Model 1: Unadjusted

Model 2: Adjusted for demographic factors: age, race/ethnicity, education, family income, and marital status.

Model 3: Adjusted for Model 2 covariates plus sexual and reproductive history: number of sexual partners, age at first intercourse, number of pregnancies, and age at menarche.

Model 4: Adjusted for Model 3 covariates plus clinical and behavioral factors: high-risk HPV infection status, contraceptive use, and alcohol consumption.

Abbreviations: CI, confidence interval; GGT, γ-glutamyltransferase; HPV, human papillomavirus; OR, odds ratio.

### Dose-response relationships

**Fig 2:**
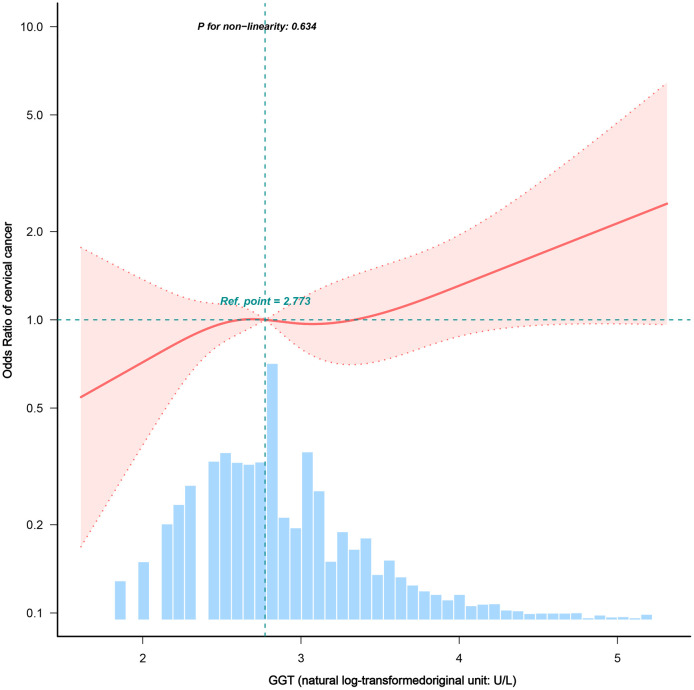
Dose-response association between serum GGT levels and cervical cancer risk. We employed a restricted cubic spline to visualize the dose – response association, graphing the adjusted odds ratio (OR; solid line) and its 95% confidence interval (shaded area) for cervical cancer across various levels of natural log-transformed GGT. The reference (OR = 1) was set to a median ln(GGT) value of 2.773. We show the dose-response relationship in the context of the population distribution of GGT levels (light blue histogram), which was obtained from models adjusted for the entire set of demographic, behavioral, and clinical covariates. **Abbreviations:** GGT, γ-glutamyltransferase; OR, odds ratio.

We identified a linear dose – response relationship between serum GGT levels and the risk of cervical cancer after accounting for confounders (P for nonlinearity = 0.634; **Fig 2**). The risk increased monotonically as the GGT levels increased.

### Subgroup analysis

**Fig 3:**
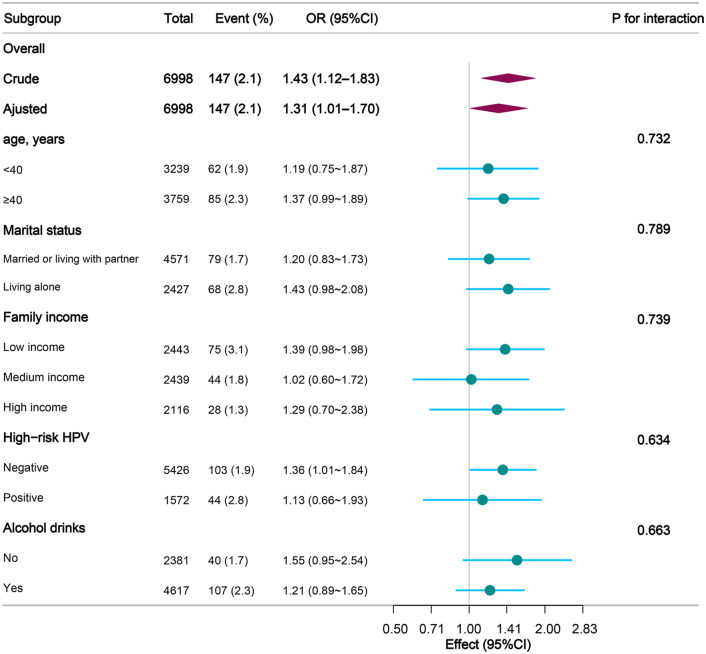
Exploratory subgroup analysis of the association between serum GGT and cervical cancer.

Exploratory subgroup analyses were performed to assess the relationship between log-transformed GGT levels and cervical cancer within the predefined strata **(**see **Fig 3**). A positive correlation was detected in most subgroups, and no significant interaction effects were identified (all P-values for interaction > 0.05). A significant positive association was observed among women with negative high-risk HPV status (OR = 1.36, 95% CI: 1.01–1.84).

Forest plot depicting the relationship between log-transformed GGT levels and cervical cancer risk across subgroups. All estimates were derived from the fully adjusted model (Model 4), and the interaction p-values were obtained from the likelihood ratio tests. **Abbreviations:** CI, confidence interval; GGT, γ-glutamyltransferase; HPV, human papillomavirus; OR, odds ratio.

### Sensitivity analysis

We utilized multiple imputation of covariates to obtain the analysis population and then replicated the multivariate regression analysis. These results demonstrate that the direction and magnitude of the relationship between GGT levels and cervical cancer were highly consistent with the outcomes of the main analysis (Supporting Information, **S1 Table**). Each log-unit increase in enzyme activity corresponded to significant risk increases across successive adjustment models (Model 6: OR = 1.30, 95% CI: 1.03–1.64; P = 0.025), sexual/reproductive adjusted model (Model 7: OR = 1.26, 95% CI: 1.00–1.59; P = 0.048), and fully adjusted model (Model 8: OR = 1.26, 95% CI: 1.00–1.59; P = 0.048). Simultaneously, the risk of cervical cancer increased by 66% when GGT was ≥ 50U/L compared to <50U/L (Model 8: OR = 1.66, 95%CI: 1.03–2.67; P = 0.038). This finding was in line with that for categorical variables in the main analysis (Model 4: OR = 1.76, 95% CI: 1.04–2.98). This series of analyses validates the reliability of our results.

We also investigated the potential impacts of BMI and tobacco exposure on these findings. After adjusting for BMI, the association between GGT levels and cervical cancer risk remained steady (Model 9: OR = 1.31, 95%CI: 1.00–1.71; P = 0.046), indicating the robustness of this relationship. Nevertheless, when we incorporated tobacco exposure into the model for adjustment, the direction of the association remained positive; however, its significance decreased and no longer reached statistical significance. At this point, the fully adjusted odds ratio for continuous GGT was 1.27 (Model 10: 95% CI, 0.97–1.66, P = 0.088), and the OR for categorical GGT was 1.69 (Model 10: 95% CI, 0.99–2.86, P = 0.053) (see Supporting Information **S2 Table**). These results imply that tobacco exposure may partly affect the association between GGT levels and cervical cancer and deserve continued attention in future research.

### Mediation analysis results

In sensitivity analyses, the relationship between GGT levels and cervical cancer was weakened and no longer statistically significant when adjusted for tobacco exposure. This finding inspired us to conduct a mediation analysis to explore the possible mechanisms of action. The results indicated that the total effect of log-GGT on the risk of cervical cancer remained statistically significant (β = 0.0014, 95%CI: 0.0001–0.0020, P = 0.046), suggesting that GGT may play a notable role in the development of cervical cancer. When decomposing the total effect, the indirect effect mediated by tobacco (serum cotinine) was statistically significant (ACME: β = 0.0003, 95% CI: 0.0001–0.0004, P < 0.001), accounting for 18.15% of the total effect (95% CI: 0.0331–0.7422). Conversely, the direct effect of GGT was not statistically significant (β = 0.0012, 95%CI: − 0.0003–0.0018, P = 0.076). See **S2 Fig** and **S3 Table** in the Supporting Information for a detailed analysis of the outcomes.

## Discussion

In this study, using multivariate regression analysis and other statistical approaches, we determined that serum GGT levels were positively associated with the risk of cervical cancer; this relationship was also observed among HPV-negative women. After adjusting for tobacco exposure, this association was no longer significant. Further mediation analysis indicated that tobacco exposure was a crucial mediator, contributing to 18.15% of the relationship between GGT and cervical cancer. This study lays the foundation for the clinical application of GGT as a potential biomarker for cervical cancer.

Via biological mechanism analysis, the relationship between serum GGT level and cervical cancer has a sound scientific foundation. GGT facilitates the production of ROS during the process of catalyzing the degradation of extracellular glutathione [9]. Previous studies have shown that ROS can promote tumor progression through various mechanisms, such as regulating immune cell function, mitochondrial metabolism, DNA methylation, and DNA damage [51]. Therefore, oxidative stress is widely considered as an important carcinogenic factor [11,52,53]. Elevated GGT levels may promote the formation of a microenvironment conducive to tumor development by increasing ROS production, such as the activation of key proinflammatory and anti-apoptotic signaling pathways, such as NF-κB [54,55]. Aberrant activation of this pathway has been shown to promote cervical cancer cell proliferation, inhibit apoptosis, and accelerate metastasis [56,57]. In addition, there is increasing evidence that chronic inflammation and persistent oxidative stress can damage the integrity of the cervical epithelial barrier, induce genomic instability in basal cells, and help establish a local immunosuppressive microenvironment, thereby promoting the occurrence of cervical cancer [58–60]. Genomic studies have shown that HPV-negative cervical cancers have different molecular features from HPV-positive tumors, typically characterized by high TP53 and CDKN2A co-mutation rates and increased PTEN mutation frequency [61,62]. This finding suggests that the development of non-HPV-related cervical cancer may depend on the actions of other carcinogenic drivers. Collectively, the oxidative stress mechanism provides a potential biological basis for the involvement of GGT in cervical carcinogenesis via non-HPV pathways. This study recognized tobacco exposure as a significant mediator in the relationship between GGT and cervical cancer. Tobacco itself has also been shown to induce systemic oxidative damage [63,64], further highlighting the key role of oxidative stress as a common biological pathway.

The exploratory subgroup analysis results indicated that even in the female population negative for high-risk HPV, the level of GGT was still positively associated with the risk of cervical cancer. Although the sample size of this subgroup was relatively limited, this finding provided preliminary evidence that GGT might be involved in the occurrence of cervical cancer through non-HPV-dependent pathways. We further emphasized that all subgroup analysis results (including the HPV-negative subgroup) were at risk of false positives, and their clinical and epidemiological significance should be interpreted with caution and not over-interpreted. At the same time, we stressed that such exploratory findings need to be verified in future studies or independent cohorts to confirm their robustness and reproducibility. In sensitivity analyses, where missing covariates were multiply imputed, the positive correlation between GGT and cervical cancer risk persisted, demonstrating the soundness of the findings. These outcomes remained stable when adjusted for covariates such as BMI. Nevertheless, after additional adjustment for tobacco exposure, the correlation between GGT levels and cervical cancer was no longer statistically significant, although the effect magnitude in the multivariate regression remained at comparable levels. Based on this, we surmise that GGT and tobacco exposure may involve overlapping biological mechanisms. Further mediation analysis revealed that tobacco exposure mediated approximately 18.15% of the total effect of GGT on cervical cancer, indicating that the impact of GGT is not confined to tobacco-related pathways, but could also influence disease risk through other independent routes. The direct effect of GGT did not attain statistical significance, which may be associated with the sample size constraint or an inherently weak effect. Collectively, these results support the use of GGT level as a potential biomarker. Future investigations can further confirm its role in the incidence and progression of cervical cancer through a prospective design, and consider the interaction effects of factors such as smoking.

The findings of this study indicate that serum GGT levels may serve as an auxiliary biomarker for cervical cancer screening. As GGT testing is straightforward and cost-effective, it might assist in identifying high-risk populations and offer more attainable health support, particularly for women in regions with limited resources. We are keenly aware of the study limitations. First, the cross-sectional design of this study could not determine the causal association between GGT levels and cervical cancer. Second, the diagnosis of cervical cancer relies on self-reporting, which may lead to inaccurate recording of information. Moreover, the small number of cervical cancer cases may have influenced the statistical power of subgroup analysis. Meanwhile, the exclusion of participants who did not complete the cancer questionnaire may have introduced selection bias. Ultimately, although the mediation analysis revealed a partial mediating effect of tobacco, the temporal sequence between GGT, smoking, and cervical cancer needs to be further elucidated. We anticipate continuing to deepen this exploration through larger-scale and longer-duration research in the future, thereby improving women’s health.

Future prospective cohort studies should be conducted to establish the temporal relationship between GGT and the development of cervical cancer, particularly its role in the development of HPV-negative cervical cancer. GGT activity should be measured directly in the cervical tissue, and its clinical value should be assessed as an auxiliary marker for HPV detection. We believe that it is essential to combine the longitudinal tracking of oxidative stress and related biomarkers to confirm the mediating effect of tobacco exposure and elucidate the direct effect of GGT.

## Conclusion

Elevated serum GGT levels were significantly associated with an increased risk of cervical cancer, and this association was statistically significant in HPV-negative individuals. Although tobacco exposure mediates this relationship to a certain extent, GGT may still be involved in the occurrence and development of cervical cancer through tobacco-independent pathways. Therefore, the clinical value of GGT as a potential adjuvant biomarker for cervical cancer requires further verification in future large-scale prospective cohort studies.

## Supporting information

S1 Fig Participant selection flowcharts for sensitivity analyses. Notes: Part 1. Flowchart for the multiple imputation sensitivity analysis. We derived an analytical sample for multiple imputation sensitivity analyses (n = 11,733) from a base cohort of 11,800 women aged 20–59 years with complete GGT and cervical cancer data. The process involved applying multiple imputations (five iterations) to handle missing covariates, followed by the exclusion of 67 participants identified as GGT outliers (values <0.5, or >99.5th percentile). Part 2. Flowchart for the complete-case sensitivity analysis of BMI and tobacco exposure. The analytic sample for this sensitivity analysis (n = 6,971) was constructed from the primary analytic sample (n = 7,039) by excluding participants with missing BMI data (n = 26), missing serum cotinine data (n = 1), and prespecified GGT outliers (n = 41). For both analyses, cervical cancer status was based on self-report. Abbreviations: BMI, body mass index; GGT, γ-glutamyltransferase; NHANES, National Health and Nutrition Examination Survey.
(TIF)

S2 Fig Mediation model of the association between serum GGT and cervical cancer through tobacco exposure. Notes: This diagram illustrates the conceptual framework for testing tobacco exposure (serum cotinine) as a mediator in the relationship between serum GGT (log-transformed) and cervical cancer. Path a represents the effect of GGT on the mediator, path b represents the effect of the mediator on cervical cancer, and path c’ represents the direct effect of GGT on cervical cancer after adjusting for the mediator. The model was adjusted for all covariates (demographic, behavioral, and clinical factors). Statistical significance of the indirect effect was evaluated using nonparametric bootstrapping with 1000 repetitions. Complete numerical results, including the effect estimates, confidence intervals, and p-values, are provided in S3 Table. Abbreviations: GGT, γ-glutamyltransferase.
(TIF)

S1 Table Association between serum GGT and cervical cancer after multiple imputation (n =  11,733). **Note:** Data are presented as OR with 95% CI intervals, and GGT levels were analyzed as a continuous variable (per 1-unit increase in the natural log-transformed value) and as a categorical variable. Model 5: Unadjusted. Model 6: Adjusted for demographic factors: age, race/ethnicity, education, family income, and marital status. Model 7: Adjusted for Model 2 covariates plus sexual and reproductive history: number of sexual partners, age at first intercourse, number of pregnancies, and age at menarche. Model 8: Adjusted for Model 3 covariates plus clinical and behavioral factors: high-risk HPV infection status, contraceptive use, and alcohol consumption. **Abbreviations:** CI, confidence interval; GGT, γ-glutamyltransferase; HPV, human papillomavirus; OR, odds ratio.
(DOCX)

S2 Table Sensitivity analysis of the serum GGT-cervical cancer association with sequential addition of body mass index and tobacco exposure. **Note:** The sensitivity analysis cohort (n = 6,971) was derived from the primary analytic sample (n = 7039) by excluding participants with missing BMI data (n = 26), missing serum cotinine data (n = 1), and prespecified GGT outliers (n = 41), as detailed in the Methods section. The results are presented as OR with 95% CI. All models were based on the fully adjusted model from the primary analysis (Table 2, Model 4) with new factors added sequentially. Model 4: Adjusted for age, race/ethnicity, education, family income, marital status, sexual and reproductive history (number of partners, age at first intercourse, gravidity, and age at menarche), high-risk HPV infection, contraceptive use, and alcohol consumption. Model 9: Adjusted for Model 4 + body mass index (BMI, categorized as normal weight [≤25 kg/m²], overweight [25–30 kg/m²], or obese [≥30 kg/m²]). Model 10: Adjusted for Model 9 + tobacco exposure (serum cotinine ≥0.05 ng/mL). **Abbreviations:** CI, confidence interval; GGT, γ-glutamyltransferase; OR, odds ratio.
(DOCX)

S3 Table Results of the mediation analysis examining tobacco exposure as a mediator in the association between serum GGT and cervical cancer. Note: The outcome was cervical cancer status. The indirect effect (average causal mediation effect) was the effect of serum GGT level on cervical cancer, which was mediated by tobacco exposure (serum cotinine). The average direct effect (ADE) was the effect of serum GGT level on cervical cancer after adjusting for the mediator. The estimates were derived from a mediation model adjusted for age, race/ethnicity, education, family income, marital status, number of sexual partners, age at first intercourse, number of pregnancies, age at menarche, high-risk HPV status, contraceptive use, and alcohol consumption. Confidence intervals and p-values for the indirect effect were based on nonparametric bootstrapping with 1000 replicates.
(DOCX)

S1 Data Minimal anonymized dataset containing the variables necessary to replicate all analyses in this study, derived from the NHANES public data (2003–2016 cycles). (CSV)

## Abbreviation

ACME: average causal mediation effect
ADE: average direct effect
BMI: body mass index
CI: confidence interval
CDC: centers for Disease Control and Prevention
GGT: γ-glutamyltransferase
HPV: human papillomavirus
NHANES: National Health and Nutrition Examination Survey
OR: odds ratio
PIR: poverty income ratio
ROS: reactive oxygen species
