# Supplementary figures and images for: Association between serum γ-Glutamyltransferase and the risk of cervical cancer: Evidence from the national health and nutrition examination survey
Source: PLoS One. 2026 Jan 2;21(1):e0339001. doi: 10.1371/journal.pone.0339001 (PMC12758775; doi:10.1371/journal.pone.0339001)

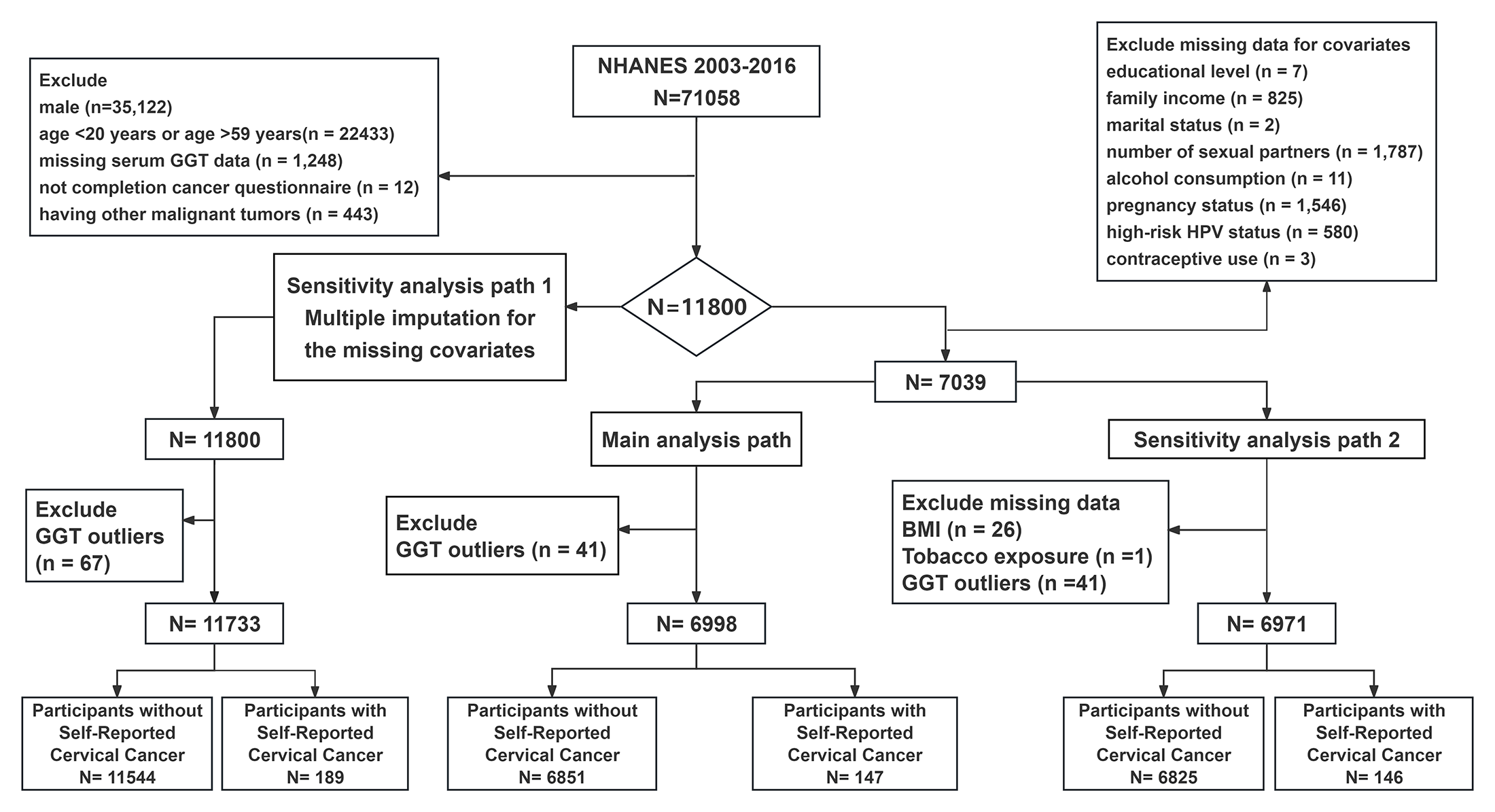

Supplement: S1 Fig — Notes: Part 1. Flowchart for the multiple imputation sensitivity analysis. We derived an analytical sample for multiple imputation sensitivity analyses (n = 11,733) from a base cohort of 11,800 women aged 20–59 years with complete GGT and cervical cancer data. The process involved applying multiple imputations (five iterations) to handle missing covariates, followed by the exclusion of 67 participants identified as GGT outliers (values <0.5, or >99.5th percentile). Part 2. Flowchart for the complete-case sensitivity analysis of BMI and tobacco exposure. The analytic sample for this sensitivity analysis (n = 6,971) was constructed from the primary analytic sample (n = 7,039) by excluding participants with missing BMI data (n = 26), missing serum cotinine data (n = 1), and prespecified GGT outliers (n = 41). For both analyses, cervical cancer status was based on self-report. Abbreviations: BMI, body mass index; GGT, γ-glutamyltransferase; NHANES, National Health and Nutrition Examination Survey. (TIF) [file pone.0339001.s001.tif]

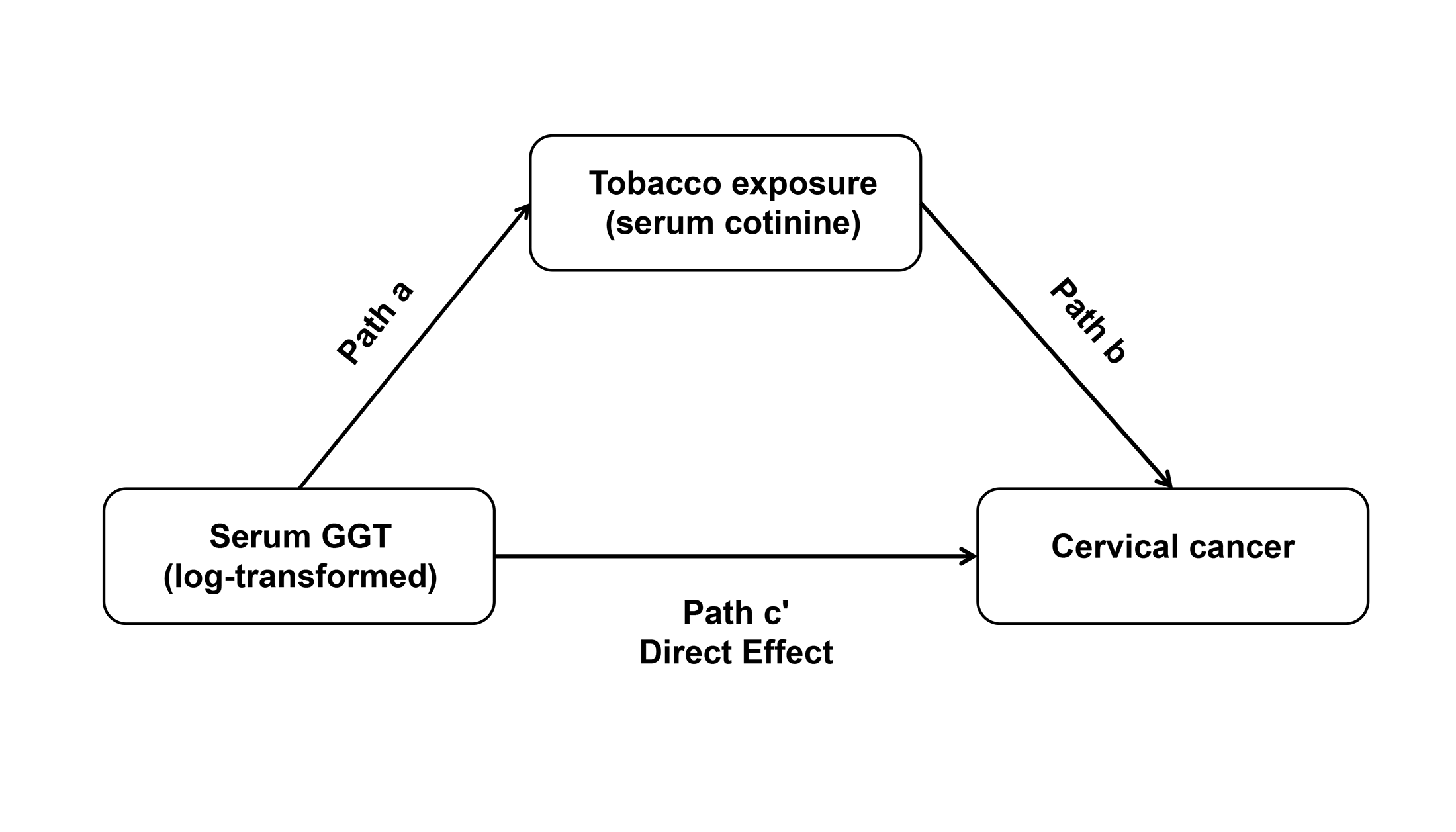

Supplement: S2 Fig — Notes: This diagram illustrates the conceptual framework for testing tobacco exposure (serum cotinine) as a mediator in the relationship between serum GGT (log-transformed) and cervical cancer. Path a represents the effect of GGT on the mediator, path b represents the effect of the mediator on cervical cancer, and path c’ represents the direct effect of GGT on cervical cancer after adjusting for the mediator. The model was adjusted for all covariates (demographic, behavioral, and clinical factors). Statistical significance of the indirect effect was evaluated using nonparametric bootstrapping with 1000 repetitions. Complete numerical results, including the effect estimates, confidence intervals, and p-values, are provided in S3 Table. Abbreviations: GGT, γ-glutamyltransferase. (TIF) [file pone.0339001.s002.tif]
