# Supplementary material for: Association between serum γ-Glutamyltransferase and the risk of cervical cancer: Evidence from the national health and nutrition examination survey
Source: PLoS One. 2026 Jan 2;21(1):e0339001. doi: 10.1371/journal.pone.0339001 (PMC12758775; doi:10.1371/journal.pone.0339001)
Supplement: S1 Table — Note: Data are presented as OR with 95% CI intervals, and GGT levels were analyzed as a continuous variable (per 1-unit increase in the natural log-transformed value) and as a categorical variable. Model 5: Unadjusted. Model 6: Adjusted for demographic factors: age, race/ethnicity, education, family income, and marital status. Model 7: Adjusted for Model 2 covariates plus sexual and reproductive history: number of sexual partners, age at first intercourse, number of pregnancies, and age at menarche. Model 8: Adjusted for Model 3 covariates plus clinical and behavioral factors: high-risk HPV infection status, contraceptive use, and alcohol consumption. Abbreviations: CI, confidence interval; GGT, γ-glutamyltransferase; HPV, human papillomavirus; OR, odds ratio. (DOCX) [file pone.0339001.s003.docx]

**S1 Table. Association between serum GGT and cervical cancer after multiple imputation (n = 11,733).**

| **Variable** | **Event,**  **n/N (%)** | **Model 5**  **OR (95% CI)** | **P value** | **Model 6**  **OR (95% CI)** | **P value** | **Model 7**  **OR (95% CI)** | **P value** | **Model 8**  **OR (95% CI)** | **P value** |
| --- | --- | --- | --- | --- | --- | --- | --- | --- | --- |
| **GGT(log)** | 189/11733 (1.6) | 1.43 (1.15–1.78) | 0.001 | 1.30 (1.03–1.64) | 0.025 | 1.26 (1.00–1.59) | 0.048 | 1.26 (1.00–1.59) | 0.048 |
| **GGT groups** |  |  |  |  |  |  |  |  |  |
| GGT <50U/L | 168/11061 (1.5) | 1(Reference) | — | 1(Reference) | — | 1(Reference) | — | 1(Reference) | — |
| GGT ≥50U/L | 21/672 (3.1) | 2.09(1.32–3.31) | 0.002 | 1.69 (1.05–2.72) | 0.030 | 1.63 (1.01–2.63) | 0.044 | 1.66 (1.03–2.67) | 0.038 |

**Note:** Data are presented as OR with 95% CI intervals, and GGT levels were analyzed as a continuous variable (per 1-unit increase in the natural log-transformed value) and as a categorical variable.

Model 5: Unadjusted.

Model 6: Adjusted for demographic factors: age, race/ethnicity, education, family income, and marital status.

Model 7: Adjusted for Model 2 covariates plus sexual and reproductive history: number of sexual partners, age at first intercourse, number of pregnancies, and age at menarche.

Model 8: Adjusted for Model 3 covariates plus clinical and behavioral factors: high-risk HPV infection status, contraceptive use, and alcohol consumption.

**Abbreviations:** CI, confidence interval; GGT, γ-glutamyltransferase; HPV, human papillomavirus; OR, odds ratio.
