# Supplementary material for: Association between serum γ-Glutamyltransferase and the risk of cervical cancer: Evidence from the national health and nutrition examination survey
Source: PLoS One. 2026 Jan 2;21(1):e0339001. doi: 10.1371/journal.pone.0339001 (PMC12758775; doi:10.1371/journal.pone.0339001)
Supplement: S2 Table — Note: The sensitivity analysis cohort (n = 6,971) was derived from the primary analytic sample (n = 7039) by excluding participants with missing BMI data (n = 26), missing serum cotinine data (n = 1), and prespecified GGT outliers (n = 41), as detailed in the Methods section. The results are presented as OR with 95% CI. All models were based on the fully adjusted model from the primary analysis (Table 2, Model 4) with new factors added sequentially. Model 4: Adjusted for age, race/ethnicity, education, family income, marital status, sexual and reproductive history (number of partners, age at first intercourse, gravidity, and age at menarche), high-risk HPV infection, contraceptive use, and alcohol consumption. Model 9: Adjusted for Model 4 + body mass index (BMI, categorized as normal weight [≤25 kg/m²], overweight [25–30 kg/m²], or obese [≥30 kg/m²]). Model 10: Adjusted for Model 9 + tobacco exposure (serum cotinine ≥0.05 ng/mL). Abbreviations: CI, confidence interval; GGT, γ-glutamyltransferase; OR, odds ratio. (DOCX) [file pone.0339001.s004.docx]

**S2 Table**.** Sensitivity analysis of the serum GGT-cervical cancer association with sequential addition of body mass index and tobacco exposure**

| **Variable** | **Primary Analysis (n=6,998)** | | **Sensitivity Analysis (n=6,971)** | | | |
| --- | --- | --- | --- | --- | --- | --- |
|  | **Model 4** | **P value** | **Model 9** | **P value** | **Model 10** | **P value** |
| **GGT(log)** | 1.31 (1.01–1.70) | 0.041 | 1.31 (1.00–1.71) | 0.046 | 1.27(0.97–1.66) | 0.088 |
| **GGT groups** |  |  |  |  |  |  |
| GGT <50U/L | 1(Reference) | — | 1(Reference) | — | 1(Reference) | — |
| GGT ≥50U/L | 1.76 (1.04–2.98) | 0.034 | 1.76 (1.04–2.98) | 0.037 | 1.69 (0.99–2.86) | 0.053 |

**Note:** The sensitivity analysis cohort (n=6,971) was derived from the primary analytic sample (n=7039) by excluding participants with missing BMI data (n=26), missing serum cotinine data (n=1), and prespecified GGT outliers (n=41), as detailed in the Methods section. The results are presented as OR with 95% CI. All models were based on the fully adjusted model from the primary analysis (Table 2, Model 4) with new factors added sequentially.

Model 4: Adjusted for age, race/ethnicity, education, family income, marital status, sexual and reproductive history (number of partners, age at first intercourse, gravidity, and age at menarche), high-risk HPV infection, contraceptive use, and alcohol consumption.

Model 9: Adjusted for Model 4 + body mass index (BMI, categorized as normal weight [<25 kg/m²], overweight [25-30 kg/m²], or obese [≥30 kg/m²]).

Model 10: Adjusted for Model 9 + tobacco exposure (serum cotinine ≥0.05 ng/mL).

**Abbreviations:** CI, confidence interval; GGT, γ-glutamyltransferase; OR, odds ratio.
