# Supplementary material for: Association between serum γ-Glutamyltransferase and the risk of cervical cancer: Evidence from the national health and nutrition examination survey
Source: PLoS One. 2026 Jan 2;21(1):e0339001. doi: 10.1371/journal.pone.0339001 (PMC12758775; doi:10.1371/journal.pone.0339001)
Supplement: S3 Table — Note: The outcome was cervical cancer status. The indirect effect (average causal mediation effect) was the effect of serum GGT level on cervical cancer, which was mediated by tobacco exposure (serum cotinine). The average direct effect (ADE) was the effect of serum GGT level on cervical cancer after adjusting for the mediator. The estimates were derived from a mediation model adjusted for age, race/ethnicity, education, family income, marital status, number of sexual partners, age at first intercourse, number of pregnancies, age at menarche, high-risk HPV status, contraceptive use, and alcohol consumption. Confidence intervals and p-values for the indirect effect were based on nonparametric bootstrapping with 1000 replicates. (DOCX) [file pone.0339001.s005.docx]

**S3 Table**. **Results of the mediation analysis examining tobacco exposure as a mediator in the association between serum GGT and cervical cancer.**

| **Effect Type** | **Estimate** | **95% Confidence Interval** | **P Value** |
| --- | --- | --- | --- |
| ****Total Effect**** | 0.0014 | 0.0001, 0.0020 | 0.046 |
| ****Direct Effect (ADE)**** | 0.0012 | -0.0003, 0.0018 | 0.076 |
| ****Indirect Effect (ACME)**** | 0.0003 | 0.0001, 0.0004 | < 0.001 |
| ****Proportion Mediated**** | 18.15% | 0.0331, 0.7422 |  |

**Note:** The outcome was cervical cancer status. The indirect effect ( average causal mediation effect) was the effect of serum GGT level on cervical cancer, which was mediated by tobacco exposure (serum cotinine). The average direct effect (ADE) was the effect of serum GGT level on cervical cancer after adjusting for the mediator. The estimates were derived from a mediation model adjusted for age, race/ethnicity, education, family income, marital status, number of sexual partners, age at first intercourse, number of pregnancies, age at menarche, high-risk HPV status, contraceptive use, and alcohol consumption. Confidence intervals and p-values for the indirect effect were based on nonparametric bootstrapping with 1000 replicates.
